# Cluster analysis and its application to healthcare claims data: a study of end-stage renal disease patients who initiated hemodialysis

**DOI:** 10.1186/s12882-016-0238-2

**Published:** 2016-03-02

**Authors:** Minlei Liao, Yunfeng Li, Farid Kianifard, Engels Obi, Stephen Arcona

**Affiliations:** KMK Consulting, Inc, 23 Headquarters Plaza, Morristown, NJ 07960 USA; Outcomes Research Methods & Analytics, US Health Economics & Outcomes Research, Novartis Pharmaceuticals Corporation, One Health Plaza, East Hanover, NJ 07936-1080 USA; Biometrics, US Medical, Novartis Pharmaceuticals Corporation, One Health Plaza, East Hanover, NJ 07936-1080 USA; Rutgers Fellow, Cardiovascular/Respiratory, US Health Economics & Outcomes Research, Novartis Pharmaceuticals Corporation, One Health Plaza, East Hanover, NJ 07936-1080 USA

**Keywords:** K-means cluster analysis, Hierarchical cluster analysis, Healthcare claims data, Cost changes

## Abstract

**Background:**

Cluster analysis (CA) is a frequently used applied statistical technique that helps to reveal hidden structures and “clusters” found in large data sets. However, this method has not been widely used in large healthcare claims databases where the distribution of expenditure data is commonly severely skewed. The purpose of this study was to identify cost change patterns of patients with end-stage renal disease (ESRD) who initiated hemodialysis (HD) by applying different clustering methods.

**Methods:**

A retrospective, cross-sectional, observational study was conducted using the Truven Health MarketScan® Research Databases. Patients aged ≥18 years with ≥2 ESRD diagnoses who initiated HD between 2008 and 2010 were included. The K-means CA method and hierarchical CA with various linkage methods were applied to all-cause costs within baseline (12-months pre-HD) and follow-up periods (12-months post-HD) to identify clusters. Demographic, clinical, and cost information was extracted from both periods, and then examined by cluster.

**Results:**

A total of 18,380 patients were identified. Meaningful all-cause cost clusters were generated using K-means CA and hierarchical CA with either flexible beta or Ward’s methods. Based on cluster sample sizes and change of cost patterns, the K-means CA method and 4 clusters were selected: Cluster 1: Average to High (*n* = 113); Cluster 2: Very High to High (*n* = 89); Cluster 3: Average to Average (*n* = 16,624); or Cluster 4: Increasing Costs, High at Both Points (*n* = 1554). Median cost changes in the 12-month pre-HD and post-HD periods increased from $185,070 to $884,605 for Cluster 1 (Average to High), decreased from $910,930 to $157,997 for Cluster 2 (Very High to High), were relatively stable and remained low from $15,168 to $13,026 for Cluster 3 (Average to Average), and increased from $57,909 to $193,140 for Cluster 4 (Increasing Costs, High at Both Points). Relatively stable costs after starting HD were associated with more stable scores on comorbidity index scores from the pre-and post-HD periods, while increasing costs were associated with more sharply increasing comorbidity scores.

**Conclusions:**

The K-means CA method appeared to be the most appropriate in healthcare claims data with highly skewed cost information when taking into account both change of cost patterns and sample size in the smallest cluster.

## Background

### Cluster analysis

Cluster analysis (CA) is a statistical technique that helps reveal hidden structures by grouping entities or objects (e.g., individuals, products, locations) with similar characteristics into homogenous groups while maximizing heterogeneity across groups [1, 2]. Entities or objects of interest are grouped together based on attributes that make them similar, with the final goal being to distinguish these entities or objects by clustering them into comparable groups and to separate them from differing groups. Conceptually, CA aims to identify cluster solutions that are relatively homogeneous within each group, leading to clusters that have high intra-class similarity, while maximizing heterogeneity between the groups, leading to low inter-class similarity across clusters. Geometrically, the objects within a cluster are close together, while the distance between clusters is further apart. CA is useful to identify groups when it is not clear which entity belongs to which group, and how many groups may best be used to cluster the entities; thus, CA helps to identify a latent structure within a dataset [1–3].

CA has been widely used in varied applications including finding a true typology, prediction based on groups, hypothesis generation, data exploration, and data reduction-or grouping similar entities into homogeneous classes, consequently organizing large quantities of information and enabling labels that facilitate communication [1, 4, 5]. Numerous specific examples of the use of CA have been reported in the literature, such as characterizing psychiatric patients on the basis of clusters of symptoms [6]; finding a group of genes that have similar biological functions [7]; or identifying medical patient groups most in need of targeted interventions [4, 5].

Less well investigated is the utility of CA in identifying macro-structures associated with changes in treatment outcomes documented in large healthcare claims databases. A particular challenge for the use of CA in healthcare claims datasets is that the distribution of healthcare expenditure data are commonly severely skewed, which complicates analyses [8, 9]. In spite of this challenge, CA may aid in identifying clusters of patients who experienced similar change in costs of care before and after treatment, and particular interest may lie in focusing attention on consistently high-cost groups or groups for whom healthcare costs dramatically increase after a change in treatment. This study employed CA to the patients with end-stage renal disease (ESRD) who were initiated on hemodialysis (HD) for their healthcare cost change patterns before and after HD and explored the feasibility of application of CA method in highly skewed claims data.

Affecting an estimated 600,000–900,000 patients in the United States, chronic kidney disease (CKD) is a complicated clinical issue increasingly recognized as both a pressing public health concern and a growing worldwide epidemic [10–15]. Kidney function progressively declines in a proportion of patients with CKD, particularly without adequate therapy. However, often, even with adequate therapy, CKD eventually progresses to devastating ESRD [16]*.* Two types of dialysis are widely used: hemodialysis (HD) and peritoneal dialysis (PD). The most common and costly of the two, HD, uses a dialysis machine and a special filter called a dialyzer to clean blood outside of the body [17, 18]. The less commonly type is PD, a procedure in which blood is cleaned inside the body via the introduction of dialysate into the abdominal cavity [18].

Even though HD is the most expensive treatment for patients with ESRD [16, 17] little has been reported beyond the aggregate level on the economic impact of the transition of ESRD patients who had previously not received dialysis to HD [19]. Hence, examining healthcare cost patterns of patients with ESRD who initiated HD and classifying these patients into groups may provide useful information to healthcare decision-makers in relation to the cost burden of HD therapy. The objectives of this analysis were: 1) to apply CA techniques to an evaluation of change in all-cause healthcare costs in patients with ESRD before and after initiating HD; 2) to explore the feasibility of application of this method to administrative claim database with highly skewed cost information; 3) to present clusters that show meaningful patterns of change of costs before and after initiating HD; and 4) to further examine these clusters to identify differences in comorbidities and other variables in the pre- and post-HD period, to see if different clinical or demographic patterns may explain the variations in overall costs across clusters.

## Methods

### Study design and data

This retrospective, cross-sectional, observational study with 2007 to 2011 data was conducted using the Truven Health Analytics’ MarketScan® Commercial Claims and Encounter and Medicare Supplemental Databases [20]. The MarketScan database, one of the most commonly used for health economics outcomes research (HEOR), is one of the largest administrative claim databases that provides healthcare costs and resource utilization in real-world settings. The databases reflect inpatient, outpatient, and outpatient prescription drug information for approximately 53 million employees and their dependents covered under commercial health insurance plans sponsored by more than 300 employers in the United States. This database provides detailed cost (payment) and healthcare utilization information for services performed in both inpatient and outpatient settings, in addition to standard demographic variables (i.e., age, sex, employment status, and geographic location). Medical claims are linked to outpatient prescription drug claims and person-level enrollment data through the use of unique enrollee identifiers [20]. The study did not require informed consent or institutional review board approval because all study data were accessed using techniques compliant with the Health Insurance Portability and Accountability Act of 1996. Thus, no identifiable protected health information was extracted during the course of the study.

### Sample selection and patient population

Patients aged ≥18 years were included in the analyses if 1) the patient had at least one confirmed diagnosis of ESRD and 2) initiated at least 2 HD sessions between 2008 and 2010. An “index date” was defined as the first HD claim within that time span. Patients were excluded if they did not have continuous enrollment for the 12 months prior to (the “pre-” HD period) or 12 months following (the “post-” HD period) the index date (pre- and post-HD periods thus may have included data from 2007 or 2011 as relevant based on index date). Patients who had a transplant or underwent PD were not excluded due to sample size and generalizability consideration. Therefore, there could be cases that patients had PD or transplant before index HD or switched to PD or had transplant after their index HD. Diagnoses were based on International Classification of Disease, Ninth Revision, Clinical Modification (ICD-9-CM) codes. Codes considered to indicate ESRD included ICD-9-CM codes 404.02, 404.12, 404.92, 404.03, 404.13, and 404.93 (hypertensive heart and CKD without heart failure and with CKD Stage V or ESRD), as well as ICD-9-CM codes 585.5 (CKD Stage 5/ESRD) and 585.6 (ESRD) (Appendix 1 includes a full set of patient medical codes that qualified a patient for inclusion in this study). Persons receiving HD were identified using Healthcare Common Procedure Coding System, Current Procedural Terminology, and ICD-9 codes, which are listed in Appendix 1 [21–23].

### Variables for clustering

The variables used for clustering were “all-cause medical costs”, or direct costs for each patient reported in the pre- and post-HD periods. All-cause medical costs included hospitalization, office, and emergency department visit costs for all purposes, including dialysis costs. Healthcare costs included payments from both insurance and out of pocket costs from patients including deductible copays and coinsurances.

### Variables for describing clusters

The variables for describing patients in clusters included gender (male or female), geographic region (Northeast, North central, South, or West), insurance type (Health Maintenance Organization [HMO] or Point-of-Service [POS] capitation, Fee-for-Service [FFS]), age (stratified as 18–24, 25–34, 35–44, 45–54, 55–64, and ≥ 65 years), and the comorbidity measures—Charlson Comorbidity Index (CCI), Elixhauser Comorbidity Index (ECI), and the Agency for Healthcare Research and Quality”s (AHRQ) top 10 Clinical Classification Software (CCS) categories. The CCI composite comorbidity score was calculated from medical records as a weighted sum of the presence of 19 documented health conditions including diabetes, peripheral vascular disease, or congestive heart failure. Weighting was accomplished by assigning a value of 1, 2, 3, or 6 to each appropriate comorbidity condition and summing these values-thus, higher values reflect greater comorbidity [24–26]. The ECI score was used to measure the burden of comorbid conditions not directly related to HD. ECI distinguishes 30 comorbid conditions identified using ICD-9-CM codes from complications by considering only secondary diagnoses unrelated to the primary diagnosis [27]. The mean ECI score for each cluster was determined; like the CCI, higher scores reflect greater comorbidity burden. The AHRQ CCS for the ICD-9-CM provides a system for classifying ICD-9-CM diagnoses or procedures into a manageable number of clinically meaningful categories. One use of the CCS method is to identify the most frequent types of conditions present in study populations. The single-level diagnosis CCS approach combines illnesses and conditions into 285 mutually exclusive categories [22, 28]. The same individual might receive a flag for as many CCS categories as the recorded diagnoses support. The CCS uses a broad definition for each disease and, unlike Charlson instruments, the CCS is reported to make little distinction regarding disease severity.

### Statistical analysis

The goal of these analyses was to cluster patients in terms of all-cause costs in the “pre” period and “post” period. Values for all-cause costs were normalized by subtracting the minimum from each value and dividing that difference by the range of all values. CA was conducted on normalized all-cause costs. Patients with similar cost patterns were “grouped” together into a set of clusters based on their costs in the pre- and post-HD period using different CA methods. Patterns of demographic information and comorbidities within each cluster were reviewed and compared/contrasted across clusters. Two major CA methods, K-means (non-hierarchical) and hierarchical CA with various linkage methods, were applied to normalized costs within the pre- and post-HD periods to identify clusters. PROC FASTCLUS and PROC CLUSTER procedures in SAS, Version 9.3, were used to conduct the cluster analyses. All other analyses were also performed using SAS, Version 9.3 [29, 30].

Several important questions must be addressed when conducting CA [1], including: What measures of similarity should be chosen to compare the entities under consideration? How should clusters be formed? And what is the optimal number of clusters? Similarity between objects is most often assessed by a distance measure, with higher values (i.e., greater distances between cases) representing greater dissimilarity between entities. Various measures are available to express similarity or dissimilarity between pairs of objects. In these analyses, we used Euclidean distance, or straight-line distance between individuals in the database-this is the most commonly used type of similarity measure when analyzing ratio or interval-scaled data [31]. Mathematically, the Euclidean distance between any 2 entities, such as B and C, with regard to 2 variables, x and y, can be expressed by the following formula [31]*:*

$$ {d}_{Euclidean}\left(B,C\right) = \sqrt{{\left({x}_B-{x}_C\right)}^2 + {\left({y}_B-{y}_C\right)}^2} $$

The values obtained from comparing all entities on both x and y (in this case, pre- and post-HD costs) form a distance matrix capturing the distances between all pairs of entities.

Clusters can be formed using either hierarchical or non-hierarchical methods. Hierarchical CA attempts to identify relatively homogenous groups of cases based on selected characteristics using an algorithm that either agglomerates or divides entities to form clusters [32]. Agglomerative algorithms begin with each entity in a separate cluster; in each subsequent step, the two clusters that are most similar are combined to build an aggregate cluster. This process is repeated until all objects are finally combined into a single cluster. Once formed, clusters cannot be split, and similarity decreases during each step. A variety of “linkage” methods may be chosen to facilitate an agglomerative algorithm and define how similar or dissimilar any two clusters may be, including, single-, complete-, or average-linkage methods, flexible beta method, McQuitty’s method, as well as the centroid method or Ward’s method (Table 1).

**Table 1:** Common agglomerative algorithms for forming clusters

Average-Linkage [39]	• The distance between 2 clusters is defined as the average distance between all pairs of the 2 clusters’ members
Centroid Method [39]	• Cluster centroids are defined as the mean values of the observation on the variables of the cluster • The distance between 2 clusters is equal to the distance between the two centroids
Single-Linkage [40–42]	• Also known as “nearest-neighbor” method • Defines similarity between clusters as the shortest distance from any one object in one cluster to any object in the other
Complete-Linkage [43]	• Also known as the “farthest-neighbor” method • Assumes the distance between 2 clusters is based on the maximum distance between any 2 members in the 2 clusters
Flexible-Beta [44, 45]	• Uses a weighted average distance between pairs of objects in different clusters to decide how far apart they are • User sets different levels of beta, and beta values less than zero optimize the dissimilarity between clusters
McQuitty’s Similarity [46]	• Assumes that each entity is a separate cluster • When two clusters are be joined, the distance of the new cluster to any other cluster is calculated as the average of the distances of the soon to be joined clusters to that other cluster • Merges together the pair of clusters that have the highest average similarity value • Continues until a specified number of clusters is found, or until the similarity measure between every pair of clusters is less than a predefined cutoff
Ward’s Method [47]	• The similarity between two clusters is the sum of squares within the clusters summed over all variables • Tends to join clusters with a small number of observations • Strongly biased toward producing clusters with the same shape and with roughly the same number of observations

In a divisive algorithm, analyses start with a single cluster containing all entities, which is then divided at each subsequent step into two additional clusters that contain the most dissimilar objects. Splitting continues until all observations are in a single-member cluster. The end product of either an agglomerative or divisive hierarchical clustering method is the construction of a hierarchy or structure depicting the formation of clusters.

The K-means method is the primary example of non-hierarchical CA. In contrast to hierarchical analyses, non-hierarchical approaches do not involve the construction of groups via iterative division or clustering; instead, they assign objects into clusters once the number of clusters is specified. To accomplish this, starting points (or cluster seeds) for each cluster must be identified, and each observation is assigned to one of the cluster seeds via some process or algorithm. In K-means CA, “*k*” points are entered into the space represented by the entities being clustered-these points represent initial group centroids [33]. The *n* observations are then partitioned into *k* clusters in which each observation belongs to the cluster with the nearest mean. Once all objects have been assigned, the positions of the *k* centroids are recalculated. These steps are repeated until the centroids no longer move, yielding a separation of the objects into groups from which the metric to be minimized can be calculated. Both hierarchical and K-means CA methods have their strengths and weakness (Table 2), and they are sometimes used in complementary fashion to converge upon an optimal cluster solution.

**Table 2:** Strengths and weaknesses of hierarchical and K-means CA methods

	Advantages	Disadvantages
Hierarchical CA	• Offers a simple yet comprehensive portrayal of clustering solutions • Measures of similarity allow this analysis to be applied to almost any type of research question • Generates an entire set of clustering solutions expediently	• Susceptible to impact of outliers in the data • Not amenable to analyzing large samples
K-means CA	• Results less susceptible to outliers in the data, influence of chosen distance measure, or the inclusion of inappropriate or irrelevant variables • Can analyze extremely large data sets	• Different solutions for each set of seed points and no guarantee of optimal clustering of observations • Not efficient when a large number of potential cluster solutions are to be considered

CA, cluster analysis

The process of conducting CA leads to a set of decisions related to the CAs performed: which method is best, and what is a reasonable number of clusters to form? In this regard, there is no right or wrong approach; ultimate consideration is given to developing a model that not only represents the data appropriately, but can be easily interpreted and understood in the context of the entities investigated-thus, successful CA requires experience and perspective to inform the selection of meaningful clusters. In this study, a final model was chosen based the following criteria: 1) In order to have a meaningful number of clusters, it was important not to have too few observations (<10) in the smallest cluster or too many small clusters; 2) As to generate a reasonable clustering pattern, it was essential to have interpretable clustering patterns; and 3) Having a reasonable number of clusters for further analysis. Selecting the number of clusters can be aided by maximizing key statistical elements of the CA: larger values of the Pseudo-F Statistic (PsF) [34] and the Cubic Clustering Criterion (CCC) [35] suggest better model fit in terms of number of clusters [29, 30, 36].

## Results

### Patients

**Fig. 1:**
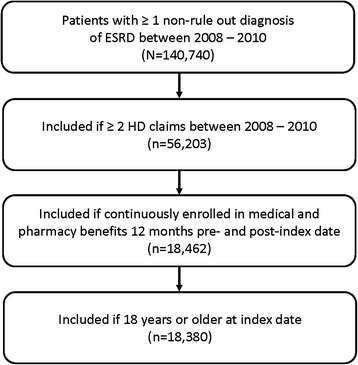
Patient selection diagram. Abbreviations: ESRD, end-stage renal disease; HD, hemodialysis

After applying the entry criteria for this study and from 140,720 individuals, a total of 18,380 individuals were identified in the MarketScan Database (Fig. 1). The average age was 63.2 years (standard deviation [*SD*] = 14.1); 46 % were aged ≥65 years, and 29 % were aged 55 to 64 years. Of the total individuals, 58 % were males, 84 % had FFS insurance plans, and 14 % had HMO or POS capitation plans. At baseline, average ECI scores were 5.8 (*SD* = 2.6) in the full sample and CCI scores were 4.6 (*SD* = 2.3); at follow-up, ECI scores had increased to 7.1 (*SD* = 3.0) while CCI scores had increased to 5.3 (*SD* = 2.4).

### Overall costs, pre- and post-HD periods

Medical costs for all patients during the pre- and post-HD periods are summarized in Table 3. We defined annual medical costs ≤ $50,000 as “average”, $50,001 to ≤ $500,000 as “high”, and > $500,000 as “very high”.

**Table 3:** All-cause medical costs in the 12-month baseline and follow-up periods

Variables	Mean (SD)	Min	Median	75th Percentile	90th^th^ Percentile	95th Percentile	99th Percentile	Max
All cause medical costs (pre-HD period)	$45,145 ($109,596)	0	$16,905	$42,758	$102,722	$178,250	$461,317	$4,771,412
All cause medical cost (post-HD period)	$48,713 ($108,506)	0	$16,330	$47,995	$123,513	$194,050	$495,240	$2,664,338

*SD* standard deviation, *Min* minimum, *Max* maximum

### Clustering techniques

Hierarchical CA with the average, centroid, single-linkage, complete-linkage, and McQuitty’s similarity methods led to cluster solutions that included clusters with unreasonable sample sizes (i.e., prone to the creation of very small clusters with <10 observations; Table 4). Both K-means CA and hierarchical CA with either the flexible-beta method or Ward’s method yielded reasonable solutions. However, the K-means solutions were more meaningful and more easily interpreted, particularly for cluster number <5, circumstances in which both Ward’s method and the flexible-beta method generated at least one cluster with large variation, which is not helpful in practice (Appendix 2, Appendix 3, and Appendix 4, respectively).

**Table 4:** Summary of results from clustering analysis methods applied

Clustering Approach	Linkage Type	Number of Clusters^a^	Cluster Sample Size (Smallest in Bold)
Hierarchical	Average	3	18,376; 3; **1**
	Average	4	18,376; 2; **1**; **1**
	Average	5	18,312; 64; 2; **1**; **1**
Hierarchical	Centroid	3	18,365; 14; **1**
	Centroid	4	18,351; 14; 14; **1**
	Centroid	5	18,351; 13; 14; **1**; **1**
Hierarchical	Single-Linkage	3	18,378; **1**; **1**
	Single-Linkage	4	18,377; **1**; **1**; **1**
	Single-Linkage	5	18,376; **1**; **1**; **1**; **1**
Hierarchical	Complete-Linkage	3	18,367; 7; **6**
	Complete-Linkage	4	18,118; 249; 7; **6**
	Complete-Linkage	5	18,118; 249; 6; 6; **1**
Hierarchical	Flexible-Beta	3	13,416; 3,732; **1232**
	Flexible-Beta	4	13,416; 3,732; 1059; **173**
	Flexible-Beta	5	8,919; 4,497; 3,732; 1,059; **173**
Hierarchical	McQuitty’s Similarity	3	18,373; 6; **1**
	McQuitty’s Similarity	4	18,367; 6; 6; **1**
	McQuitty’s Similarity	4	18,205; 162; 6; 6; **1**
Hierarchical	Ward’s Method	3	15,718; 2,315; **347**
	Ward’s Method	4	15,718; 2,315; 284; **63**
	Ward’s Method	5	15,718; 2,315; 239; 63; **45**
Non-hierarchical	N/A	3	336; 17,909; **135**
	N/A	4	113; 16,624; 1,554; **89**
	N/A	5	116; 594; 16,162; **48**; 1,460

*N/A* not applicable. ^a^Number of clusters in the model

**Fig. 2:**
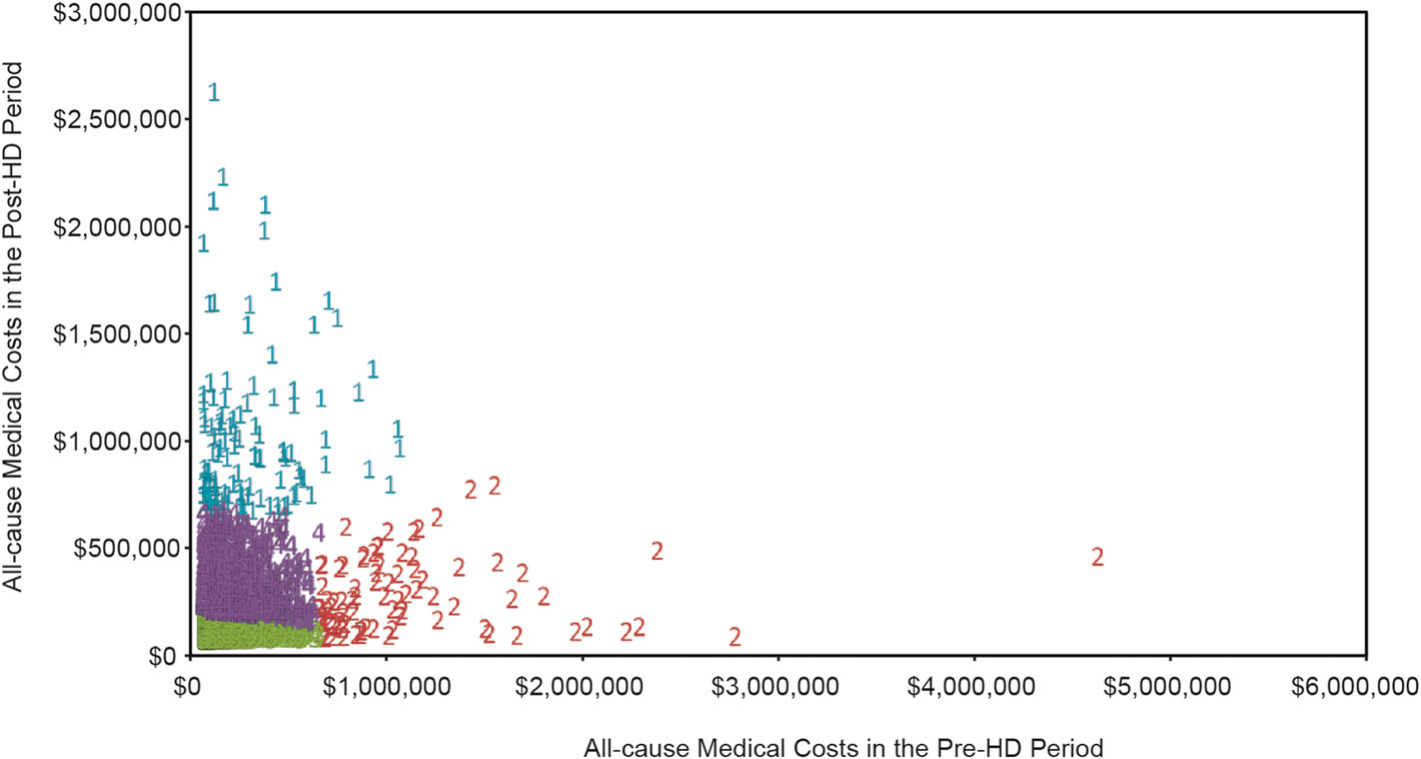
Scatter plot by cluster of all-cause medical costs in pre- and post-HD periods by K-means CA with four cluster solutions^a^. Footnote: ^a^Pseudo F Statistics = 13,979.98; Approximate Expected Over-All *R* ^*2*^ = 0.79; Cubic Clustering Criterion = −63.93. Each cluster is labeled by corresponding number

**Fig. 3:**
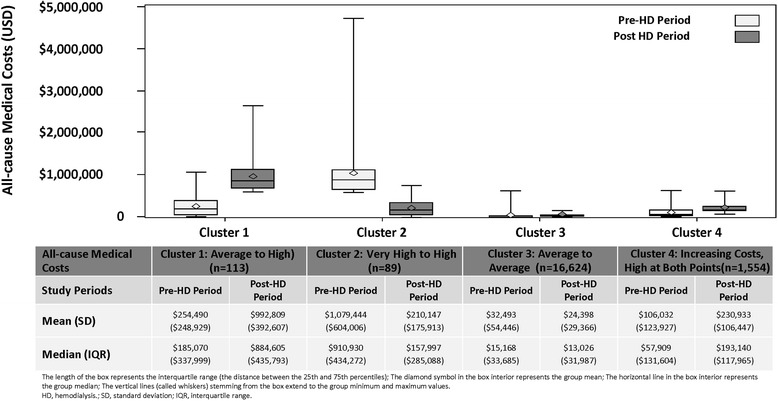
All-cause medical costs in pre- and post-HD periods by cluster^a^

Upon inspection, the best K-means solution included 4 clusters (Fig. 2). More formal criteria associated with each of the K-means solutions suggested 4 clusters yielded maximum separation between clusters (4-cluster solution: *PsF* = 13,979.98; *CCC* = −63.928 compared with *PsF* = 10,502.25 and *CCC* = −99.702 for a 3-cluster solution, and *PsF* = 13,109.62 and *CCC* = −70.634 for a 5-cluster solution). Empirically, the 4-cluster solution was judged to be more appropriate and more easily interpretable than either the 3- or 5-cluster solution. Thus, a 4-cluster K-means solution was chosen for further investigation (Fig. 3). The 4 clusters in this model included a cluster with average costs pre-HD and high costs post-HD (Cluster 1: Average to High); a cluster (the smallest) with very high costs in the pre-HD period and high costs in the post-HD period, along with a substantial decrease in average cost from pre- to post-HD (Cluster 2: Very High to High); a group (the largest) exhibiting average costs in both the pre- and post-HD periods, with a small decrease in average costs from baseline to follow-up (Cluster 3: Average to Average); and finally, the second largest group, exhibiting “high” costs in both the pre- and post-HD periods, along with relatively sizeable cost increases from baseline to follow-up (Cluster 4: Increasing Costs, High at Both Points). Figure 3 and its corresponding table summarize the cost changes in the 12-month pre- and post-HD periods, respectively. Cluster 1 (Average to High) reveals median costs that increased from $185,070 to $884,605; Cluster 2 (Very High to High) shows that the median costs decreased from $910,930 to $157,997; Cluster 3 (Average to Average) reports that the median costs were relatively stable and remained low from $15,168 to $13,026, and Cluster 4 (Increasing Costs, High at Both Points) reveals that the median costs increased from $57,909 to $193,140.

Basic demographic information and clinical characteristics of the sample divided into the four clusters suggested by K-means analysis are summarized in Table 5; the top 10 CSS disease categories in the baseline and follow-up period for each cluster are reflected in Appendix 5. Patients in Cluster 3 (Average to Average) (i.e., those with stable average costs before and after initiating HD) tended to be older, with an average age of 63.9 years compared with an average age of 55.5 through 57.6 years in the other three clusters. Otherwise, there was little to no meaningful difference across each cluster in terms of gender, living region, or health insurance type (Table 5). Economically, Clusters 1 (Average to High) and 4 (Increasing Costs, High at Both Points) were both associated with increasing costs from pre- to post-HD. Clinically, substantial increases in comorbidity scores, including both the ECI and the CCI, were observed from baseline to the follow-up period in both these groups. In contrast, Cluster 2 (Very High to High) experienced a reduction in costs after starting HD, from very high to high costs, and both ECI and CCI scores were relatively stable after initiating HD. In addition, relatively stable ECI and CCI scores were reported in Cluster 3, where stable average costs before and after HD were identified. Cluster 3 (Average to Average) exhibited notably low comorbidity scores during the post-HD period when compared with the three other clusters (Table 5).

**Table 5:** Demographic and clinical characteristics of patients grouped into 4 proposed clusters using K-means CA

	Cluster 1: Average to High		Cluster 2: Very High to High		Cluster 3: Average to Average		Cluster 4: Increasing Costs, High at Both Points	
	(*n* = 113)		(*n* = 89)		(*n* = 16,624)		(*n* = 1554)	
Age (y), mean (SD)	57.6 (11.6)		55.5 (14.8)		63.9 (14.0)		56.2 (12.8)	
Age (y), n (%)								
18-24	0 (0.0)		4 (4.5)		121 (0.7)		33 (2.1)	
25-34	2 (1.8)		7 (7.9)		355 (2.1)		54 (3.5)	
35-44	15 (13.3)		6 (6.7)		1026 (6.2)		156 (10.0)	
45-54	24 (21.2)		19 (21.3)		2401 (14.4)		375 (24.1)	
55-64	50 (44.2)		33 (37.1)		4652 (28.0)		609 (39.2)	
65+	22 (19.5)		20 (22.5)		8069 (48.5)		327 (21.0)	
Sex, n (%)								
Male	66 (58.4)		48 (53.9)		9599 (57.7)		924 (59.5)	
Female	47 (41.6)		41 (46.1)		7025 (42.3)		630 (40.5)	
Region in the United States, n (%)								
Northeast	12 (10.6)		12 (13.5)		1843 (11.1)		192 (12.4)	
North central	32 (28.3)		18 (20.2)		6084 (36.6)		444 (28.6)	
South	38 (33.6)		39 (43.8)		6354 (38.2)		625 (40.2)	
West	30 (26.5)		19 (21.3)		2235 (13.4)		286 (18.4)	
Unknown	1 (0.9)		1 (1.1)		108 (0.6)		7 (0.5)	
Health insurance type, n (%)								
FFS	87 (77.0)		71 (79.8)		13,967 (84.0)		1237 (79.6)	
HMO and POS capitation	20 (17.7)		17 (19.1)		2304 (13.9)		270 (17.4)	
Missing	6 (5.3)		1 (1.1)		353 (2.1)		4 (3.0)	
Comorbidity Score Indices^a^	Pre-HD Period	Post-HD Period	Pre-HD Period	Post-HD Period	Pre-HD Period	Post-HD Period	Pre-HD Period	Post-HD Period
ECI, mean (SD)	6.9 (3.4)	10.8 (3.5)	9.0 (4.1)	9.3 (3.7)	5.7 (2.5)	6.8 (2.8)	6.5 (3.0)	9.5 (3.2)
CCI, mean (SD)	5.0 (2.7)	7.1 (2.4)	5.6 (3.2)	6.2 (2.9)	4.6 (2.2)	5.1 (2.3)	5.0 (2.5)	6.5 (2.7)

*FFS* fee-for-service, *HD*, hemodialysis, *HMO* health maintenance organization, *PPS*, point-of-service, *ECI* elixhauser comorbidity index, *CCI* charlson comorbidity index, *SD* standard deviation. ^a^Identification was based on non-rule out diagnosis

## Discussion

In this retrospective observational analysis of claims data from commercially insured ESRD patients initiating HD, CA successfully revealed a latent structure underlying all-cause cost data before and after the start of HD. Several clustering techniques were applied, including both K-means CA and a set of hierarchical clustering analyses with multiple agglomerative algorithms that included average, centroid, single- and complete-linkage methods; McQuitty’s similarity method; and both the flexible-beta and Ward’s methods. Models generated by both K-means and hierarchical cluster CA with flexible beta and Ward’s methods produced clusters of reasonable sample size. K-means CA yielded the most informative categorization of patients generating more reasonable clusters from a practical perspective than did the other statistical methods. In addition, the K-means solutions were the most easily interpreted. In contrast, Ward’s and the flexible-beta methods led to solutions with at least one cluster with large variability (or spread), which can be difficult to interpret. Among the models suggested by K-means CA, a 4-cluster solution appeared to be the most appropriate for these data: associated criteria suggested a 4-cluster solution offers maximum separation of clusters compared with either a 3- or 5-cluster solution. In addition, a 4-cluster solution was more interpretable, and thus more appropriate to apply than other methods.

Mean all-cause medical costs in this sample of privately insured patients ranged from approximately $45,000 (USD) prior to the initiation of HD to $49,000 (USD) after; median costs ranged from $17,000 in the 12 months before HD initiation to $16,000 in the 12 months following HD initiation. Interestingly, these reported costs are generally lower than those found in other analyses in other populations. In 2004, the average annual Medicare expenditure for an ESRD patient started on HD was reported to be $72,000 (USD) [37], increasing to $77,500 (USD) in 2012 [11]. Other estimates suggest annual all-cause costs for HD patients to be as high as $174,000 (USD) in a privately insured population [17]. It is worth noting that the current results reflect payment from insurance claims made in the “real-world setting”. Importantly, a switch to HD from no dialysis in the present data set was only associated with a modest increase in average and median annual costs for ESRD patients on the whole, suggesting that the transition to HD does not generally add substantial costs to average annual care for a patient and may be associated with quite similar costs for the majority of late-stage patients with renal disease in comparison to their cost of care immediately before initiating HD. It is interesting to note that in both the pre- and post-HD assessment periods, 75 % of patients had costs below the average of $45,000 and $49,000 (USD), respectively-thus, it appears as if a relatively small fraction of patients are driving up the overall increase in costs after initiating HD, a contention supported by CA.

More specifically, CA demonstrated that the data could be reasonably represented by 4 clusters of patients: those with average costs before and after initiating HD (90 % of the full sample); those with high costs before and high/increased costs after (8 %); those with average costs who incur high costs after initiating HD (0.6 %); and a cluster with very high costs prior to initiating HD who see their annual costs reduced to a high level (0.5 %). Thus, overall costs stay stable for most ESRD patients initiating HD, suggesting transition to HD *per se* is not an important driver of cost for the majority of patients. A minority of patients drive an increase in overall costs after HD initiation.

Because of the different cost patterns in each group, it is worthwhile to better understand patients in each cluster to help predict and contain the costs of HD. Comorbidities seem to be particularly relevant to costs, with increasing comorbidity scores from baseline to follow-up periods in those clusters associated with an increase in costs during follow-up, and more stable comorbidity scores associated with more stable costs (or even declining costs). This is consistent with other research: one study demonstrated that an increased level of comorbidity was associated with higher cost in the 2 years prior to starting HD [13], while another demonstrated a clear relationship between CCI scores and costs [38]. These data suggest timely management of comorbidities or the prevention of comorbidities may be critical for containing costs in patients starting HD. Interestingly, the older age of the patients in the most stable cost cluster (i.e., Cluster 3) suggests that there may be a difference in expression of ESRD in these patients compared with the other clusters, perhaps a factor that manifests itself as both a later-in-life need for HD as well as better overall health (e.g., fewer comorbidities).

In aggregate, costs are high at an absolute level, both before and after the initiation of HD, suggesting that the healthcare costs of the majority of ESRD patients not treated with HD are not substantially lower than the costs of care for these patients immediately after starting HD. Thus, HD does not add substantial costs for most patients and seems like an economically feasible option in most patients with CKD, given the overall high cost of care for these patients prior to initiating HD. True cost containment for patients with ESRD likely requires more aggressive or widespread intervention before patients reach this advanced stage of disease, where costs are high before and after HD. One overall strategy that may reduce costs includes early referral to a nephrologist in the period before starting HD [16]. HD is not an important cost driver for the majority of patients, so limiting HD may not contain costs for these patients. There is a need to better understand the fraction of the population that is driving higher post-HD costs, and consider ways to mitigate the costs associated with their transition to HD.

### Limitations

Interpretation of these results must be informed by limitations of these analyses. First, these analyses were conducted only in those employed individuals with commercial insurance coverage and some individuals with Medicare coverage; thus, these results from a relatively healthy population may not be fully generalized to individuals with Medicare, Medicaid, other insurance, and no insurance. Second, administrative claims data cannot capture deaths and changes of employment; therefore, the cost not captured due to loss to follow-up may lead to selection bias. In addition, administrative claims data are not collected for research purposes and measurement error may have been introduced by coding that was in error or driven by reimbursement needs more so than research needs. Further, administrative claims data does not collect clinical information that would have been valuable additions to these analyses, such as laboratory test results or vital signs. Access to patients’ claims prior to their enrollment in MarketScan databases is not available. Retrospective analysis limits the study to those who are clinically diagnosed and incur health care resource utilization through claims; resource utilization not identified by claims would not be included in these analyses. Finally, treatment costs in future studies should examine what cost drivers may have influenced increases or decreases in costs for each cluster.

## Conclusions

CA was a useful statistical technique for evaluating a claims data set that included skewed healthcare cost data. One implication of these analyses is that costs for most patients with ESRD stay relatively stable after starting HD; a minority of patients drive overall increasing annual costs after initiation of dialysis. These increasing costs may be driven, in part, by a greater comorbidity burden among these patients.

## Figures and Tables

**Fig. 4:**
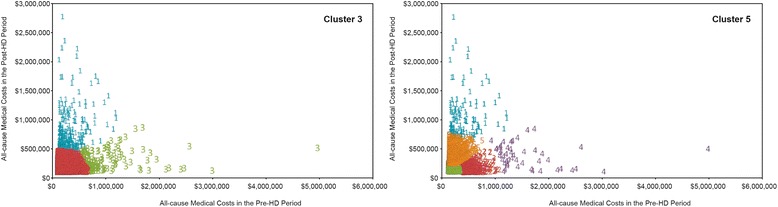
Scatter plots of all-cause medical costs in pre- and post-HD periods by K-means CA with 3- and 5-cluster solutions^a^. ^a^Each cluster is labeled by corresponding number

**Fig. 5:**
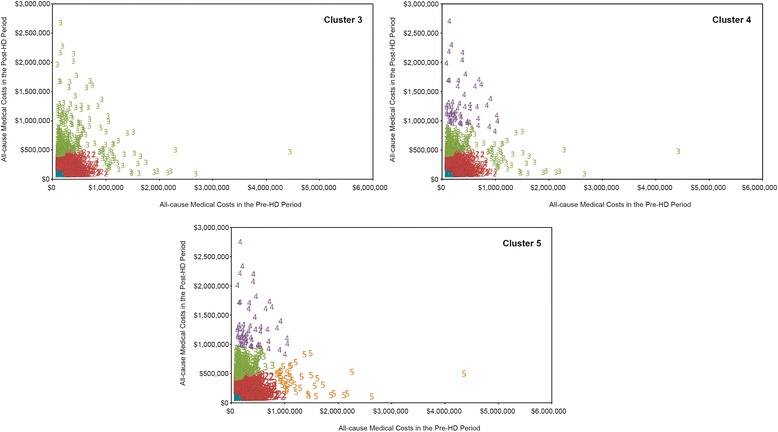
Scatter plots of all-cause medical costs in pre- and post-HD periods by Hierarchical CA with Ward Method and 3-, 4-, and 5-cluster solutions. ^a^Each cluster is labeled by corresponding number

**Fig. 6:**
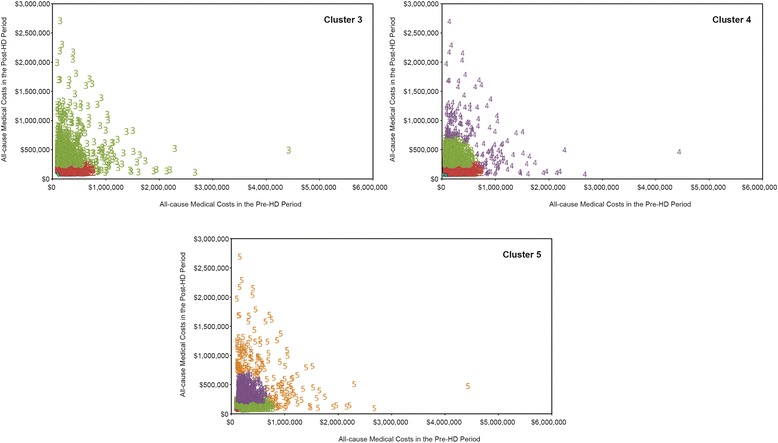
Scatter plots of all-cause medical costs in pre- and post-HD periods s by Hierarchical CA with Flexible-Beta Method and 3-, 4-, and 5-cluster solutions^a^. ^a^Each cluster is labeled by corresponding number

## Appendix 1

**Table 6:** Medical codes indicating HD

Code	Description	Source
458.21	Hypotension from HD	ICD-9-CM diagnosis
V56.31	Adequacy testing for HD	ICD-9-CM diagnosis
39.95	HD	ICD-9-CM procedure
A4680	Activated carbon filter for HD (each)	HCPCS
A4690	Dialyzer (artificial kidneys); all types and all sizes for HD	HCPCS
A4706	Bicarbonate concentrate solution per gallon for HD	HCPCS
A4707	Bicarbonate concentrate powder per packet for HD	HCPCS
A4708	Acetate concentrate solution per gallon for HD	HCPCS
A4709	Acid concentrate solution per gallon for HD	HCPCS
A4730	Fistula cannulation set for HD	HCPCS
A4740	Shunt accessory for HD (any type, each)	HCPCS
A4750	Blood tubing, arterial or venous for HD (each)	HCPCS
A4755	Blood tubing, arterial and venous combined for HD (each)	HCPCS
A4802	Protamine sulphate per 50 mg for HD	HCPCS
A4870	Plumbing and/or electrical work for home HD equipment	HCPCS
A4890	Contracts, repair, and maintenance for HD equipment	HCPCS
A4918	Venous pressure clamp for HD (each)	HCPCS
E1520	Heparin infusion pump for HD	HCPCS
E1530	Air bubble detector for HD (each, replacement)	HCPCS
E1540	Pressure alarm for HD (each, replacement)	HCPCS
E1550	Bath conductivity meter for HD (each)	HCPCS
E1560	Blood leak detector for HD (each, replacement)	HCPCS
E1575	Transducer protectors/fluid barriers for HD	HCPCS
E1580	Unipuncture control system for HD	HCPCS
E1590	HD machine	HCPCS
E1600	Delivery and/or installation charges for HD equipment	HCPCS
E1610	Reverse osmosis water purification system for HD	HCPCS
E1615	Deionizer water purification system for HD	HCPCS
E1620	Blood pump replacement for HD	HCPCS
E1625	Water-softening system for HD	HCPCS
E1636	Sorbent cartridges for HD	HCPCS
G0365	Vessel mapping of vessels for HD access	HCPCS
G0392	Transluminal balloon angioplasty, percutaneous, for maintenance of hemodialysis access, arteriovenous fistula or graft, arterial	HCPCS
G0393	Transluminal balloon angioplasty, percutaneous for maintenance of HD access, arteriovenous fistula or graft, venous	HCPCS
G8081	ESRD patient requiring HD vascular access documented to have received autogenous AV fistula	HCPCS
G8082	ESRD patient requiring HD vascular access documented to have received vascular access other than autogenous AV fistula	HCPCS
G8085	ESRD patient requiring hemodialysis vascular access was not candidate for autogenous arteriovenous fistula	HCPCS
S9335	Home therapy for HD	HCPCS
90935	HD procedure with single evaluation by a physician or other qualified health care professional	CPT
90937	HD procedure requiring repeated evaluation(s) with or without substantial revision of dialysis prescription	CPT
90940	HD access flow study to determine blood flow in grafts and arteriovenous fistulae by an indicator method	CPT
93990	Duplex scan of HD access	CPT
36800	Insertion of cannula for other purpose for HD (separate procedure); vein to vein	CPT
36810	Insertion of cannula for other purpose for HD (separate procedure); arteriovenous, external	CPT
36815	Insertion of cannula for other purpose for HD (separate procedure); arteriovenous, external revision, or closure	CPT

*HD* hemodialysis, *ESRD* end-stage renal failure, *HCPCS* healthcare common procedure coding system, *CPT* current procedural terminology, *ICD-9-CM* International Classification of Disease, 9th Revision, clinical modification

## Appendix 5

**Table 7:** Top 10 CSS disease categories in the pre- and post-HD periods

Cluster and Descriptive Costs (n)	CCS Disease Categories in the Pre-HD Period (%)	CCS Disease Categories in the Post-HD Period (%)
Cluster 1: Average to High (*n* = 113)	1. Acute and unspecified ESRD (82 %) 2. CKD (67 %) 3. Essential hypertension (61 %) 4. Other lower respiratory disease (60 %) 5. Other connective tissue disease (60 %) 6. Deficiency and other anemia (58 %) 7. Residual codes; unclassified (57 %) 8. Type 2 diabetes without complication (56 %) 9. Other circulatory disease (54 %) 10. Cardiac dysrythmias (52 %)	1. CKD (99 %) 2. Acute and unspecified ESRD (90 %) 3. Deficiency and other anemia (89 %) 4. Septicemia (except in labor) (89 %) 5. Complication of device, implant, or graft (86 %) 6. Residual codes; unclassified (83 %) 7. Respiratory failure; insufficiency; arrest (adult) (82 %) 8. Other circulatory disease (80 %) 9. Other lower respiratory disease (78 %) 10. Cardiac dysrhythmias (76 %)
Cluster 2: Very High to High (*n* = 89)	1. Acute and unspecified ESRD (87 %) 2. Other lower respiratory disease (80 %) 3. Respiratory failure; insufficiency; arrest (78 %) 4. Residual codes; unclassified (75 %) 5. Cardiac dysrythmias (73 %) 6. Essential hypertension (70 %) 7. Septicemia (67 %) 8. Deficiency and other anemia (65 %) 9. CKD (65 %) 10. Other circulatory disease (64 %)	1. CKD (97 %) 2. Acute and unspecified ESRD (89 %) 3. Deficiency and other anemia (85 %) 4. Respiratory failure; insufficiency; arrest (78 %) 5. Residual codes; unclassified (78 %) 6. Complication of device, implant or graft (76 %) 7. Essential hypertension (75 %) 8. Other aftercare (73 %) 9. Other lower respiratory disease (72 %) 10. Other connective tissue disease (72 %)
Cluster 3: Average to Average (*n* = 16,624)	1. CKD (92 %) 2. Deficiency and other anemia (69 %) 3. Essential hypertension (60 % 4. Hypertension with complications and secondary hypertension (59 %) 5. Acute and unspecified ESRD (57 %) 6. Type 2 diabetes without complications (475) 7. Type 2 diabetes with complications (42 %) 8. Residual codes; unclassified (41 %) 9. Other lower respiratory disease (40 %) 10. Complication of device; implant or graft (37 %)	1. CKD (100 %) 2. Deficiency and other anemia (86 %) 3. Complication of device; implant or graft (73 %) 4. Hypertension with complications and secondary hypertension (70 %) 5. Essential hypertension (62 %) 6. Acute and unspecified ESRD (55 %) 7. Other disease of kidney and ureters (53 %) 8. Type 2 diabetes without complication (50 %) 9. Residual codes; unclassified (50 %) 10. Type 2 diabetes with complications (45 %)
Cluster 4: Increasing Costs, High at Both Points (*n* = 1554)	1. CKD (85 %) 2. Acute and unspecified ESRD (70 %) 3. Deficiency and other anemia (66 %) 4. Essential hypertension (65 %) 5. Hypertension with complications and secondary hypertension (58 %) 6. Residual codes; unclassified (52 %) 7. Type 2 diabetes without complication (52 %) 8. Other lower respiratory disease (51 %) 9. Type 2 diabetes with complications (50 %) 10. Fluid and electrolyte disorders (45 %)	1. CKD (92 %) 2. Deficiency and other anemia 3. Hypertension with complications and secondary hypertension 4. Complication of device; implant or graft 5. Residual codes; unclassified 6. Acute and unspecified ESRD 7. Essential hypertensions 8. Other aftercare 9. Other circulatory disorder 10. Other lower respiratory disease

*ESRD* end-stage renal disease, *CKD* chronic kidney disease
